# Endoplasmic Reticulum–Mitochondrial Ca^2+^ Fluxes Underlying Cancer Cell Survival

**DOI:** 10.3389/fonc.2017.00070

**Published:** 2017-05-03

**Authors:** Hristina Ivanova, Martijn Kerkhofs, Rita M. La Rovere, Geert Bultynck

**Affiliations:** ^1^Laboratory of Molecular and Cellular Signaling, Department of Cellular and Molecular Medicine, Leuven Kanker Instituut (LKI), KU Leuven, Leuven, Belgium

**Keywords:** cancer, endoplasmic reticulum–mitochondrial Ca^2+^ fluxes, Ca^2+^ signaling, inositol 1,4,5-trisphosphate receptor/Ca^2+^ channels, cell cycle regulation, mitochondrial metabolism, cell death signaling

## Abstract

Calcium ions (Ca^2+^) are crucial, ubiquitous, intracellular second messengers required for functional mitochondrial metabolism during uncontrolled proliferation of cancer cells. The mitochondria and the endoplasmic reticulum (ER) are connected *via* “mitochondria-associated ER membranes” (MAMs) where ER–mitochondria Ca^2+^ transfer occurs, impacting the mitochondrial biology related to several aspects of cellular survival, autophagy, metabolism, cell death sensitivity, and metastasis, all cancer hallmarks. Cancer cells appear addicted to these constitutive ER–mitochondrial Ca^2+^ fluxes for their survival, since they drive the tricarboxylic acid cycle and the production of mitochondrial substrates needed for nucleoside synthesis and proper cell cycle progression. In addition to this, the mitochondrial Ca^2+^ uniporter and mitochondrial Ca^2+^ have been linked to hypoxia-inducible factor 1α signaling, enabling metastasis and invasion processes, but they can also contribute to cellular senescence induced by oncogenes and replication. Finally, proper ER–mitochondrial Ca^2+^ transfer seems to be a key event in the cell death response of cancer cells exposed to chemotherapeutics. In this review, we discuss the emerging role of ER–mitochondrial Ca^2+^ fluxes underlying these cancer-related features.

## Mitochondrial Metabolism in Cancer Cell Survival

Cell proliferation requires an increased supply of nutrients, like glucose and glutamine, to achieve a balance between biomass and energy production for making new cells (1). Glucose, the major source of macromolecular precursors and ATP generation, is transformed into pyruvate *via* the cytosolic process glycolysis. In aerobic conditions, pyruvate is transported into the mitochondria and metabolized to CO_2_ through the tricarboxylic acid (TCA) cycle. The TCA cycle is coupled to oxidative phosphorylation (OXPHOS), which is a pathway for the production of large amounts of ATP. In contrast, in anaerobic conditions, pyruvate is fermented to lactate, a process often referred to as anaerobic glycolysis, which is less energy effective. Nevertheless, proliferative cells exhibit enhanced glycolysis, producing high levels of lactate, even in the presence of O_2_ (aerobic glycolysis) (2). Cancer cells, which are characterized by uncontrolled proliferation and suppressed apoptosis, tend to switch to aerobic glycolysis despite the presence of sufficient O_2_ to support the OXPHOS pathway. As such, these cells display an elevated glucose consumption albeit without a proportional increase in its oxidation to CO_2_ together with an increased lactate production and lactate export, a phenomenon known as “Warburg effect” (3–5). Although glycolysis can produce ATP at a faster rate than OXPHOS (6) and may fuel biosynthesis with intermediates, cancer cells do not rely purely on glycolysis. The reprogrammed cellular metabolism in tumors also maintains sufficient levels of OXPHOS by using pyruvate generated by glycolysis. Indeed, the TCA cycle appears to complement glycolysis, supplying enough ATP, NADH, and biomass precursors for the biosynthesis of other macromolecules, like phospholipids and nucleotides (7). For instance, the TCA cycle intermediate oxaloacetate is used as a substrate for the biosynthesis of uridine monophosphate, a precursor of uridine-5′-triphosphate and cytidine triphosphate involving a rate-limiting step executed by dihydroorotate dehydrogenase, which, in turn, catalyzes the *de novo* synthesis of pyrimidines in the inner mitochondrial membrane (8). Its dehydrogenase activity depends on the electron transport chain (ETC), where it feeds the electrons of the dihydroorotate oxidation to the ETC by reducing respiratory ubiquinone. Thus, adequate ETC activity and proper pyrimidine biosynthesis are intimately linked (8).

## Mitochondrial Ca^2+^ Signals as Regulators of Cell Death and Survival

Ca^2+^, a cofactor of several rate-limiting TCA enzymes [pyruvate-, isocitrate-, and α-ketoglutarate dehydrogenases (PDH, IDH, and αKGDH)], plays a pivotal role in the regulation of mitochondrial metabolism and bioenergetics (9). As such, Ca^2+^ present in the mitochondrial matrix is required for sufficient NADH and ATP production (10).

### Transfer of Ca^2+^ Signals from the Endoplasmic Reticulum (ER) to the Mitochondria

The accumulation of Ca^2+^ into the mitochondria strictly depends on the ER, which serves as the main intracellular Ca^2+^-storage organelle. Ca^2+^ is stored in the ER by the action of ATP-driven sarco/endoplasmic reticulum Ca^2+^-ATPase (SERCA) with SERCA2b (11) as the housekeeping isoform and by ER luminal Ca^2+^-binding proteins like calreticulin and calnexin (12). Ca^2+^ can be released from the ER *via* intracellular Ca^2+^-release channels, including inositol 1,4,5-trisphosphate receptors (IP_3_Rs) and ryanodine receptors (RyRs). IP_3_Rs, which are activated by the second messenger IP_3_, are ubiquitously expressed in virtually all human cell types (13, 14). IP_3_ is produced through the hydrolysis of phosphatidyl inositol 4,5-bisphosphate by phospholipase C (PLC)β/γ, an enzyme activated in response to hormones, neurotransmitters, and antibodies. IP_3_R activity can be suppressed by compounds like xestospongin B (15), which directly inhibits IP_3_Rs, or U73122, which inhibits PLC activity (16). Although 2-APB (17) and xestospongin C (18) are also used as IP_3_R inhibitors, these compounds affect other Ca^2+^-transport systems. For instance, 2-APB is known to inhibit store-operated Ca^2+^ entry through Orai1 (19) and SERCA (20), and to activate Orai3 channels (19). In addition, similarly to its analogs like DPB162-AE, 2-APB can induce a Ca^2+^ leak from the ER, partially mediated by ER-localized Orai3 channels (20–23). Xestospongin C also inhibits SERCA with a potency that is equal to its inhibitory action on IP_3_Rs (24). RyRs are predominantly expressed in excitable cells, including several muscle types, neuronal cells, and pancreatic β cells (25). In most cells, RyRs are mainly activated by cytosolic Ca^2+^ *via* Ca^2+^-induced Ca^2+^ release, while in skeletal muscle they are activated through a direct coupling with the dihydropyridine receptor upon depolarization (26). RyR activity can be counteracted by dantrolene (27) and high concentrations of ryanodine (28).

The efficient Ca^2+^ exchange between the ER and the mitochondria takes place in specialized microdomains, which are established by organellar contact sites and which can be isolated biochemically as mitochondria associated-ER membranes (MAMs) (29–31). Several proteins are involved in ER–mitochondrial tethering, including IP_3_Rs at the ER side and the Ca^2+^-permeable channels voltage-dependent anion channel type 1 (VDAC1) at the mitochondrial side (32, 33). The Ca^2+^ released through IP_3_Rs and eventually transferred to the mitochondrial intermembrane space by VDAC1 accumulates in the mitochondrial matrix *via* the mitochondrial Ca^2+^ uniporter (MCU). The functional properties of the MCU are tightly regulated by a growing list of interacting proteins, which enable a tight control over the Ca^2+^ levels in the mitochondrial matrix (34). These MCU modulators have an important cell physiological impact on mitochondrial metabolism, cell survival, and cell death (35–44).

Seminal work using aequorin targeted to the mitochondria revealed that IP_3_-evoked Ca^2+^ signals were efficiently transferred into the mitochondria even when IP_3_-induced cytosolic Ca^2+^ concentration ([Ca^2+^]_cyt_) rises were relatively small (45). In contrast, artificial rises in [Ca^2+^]_cyt_ were ineffective in increasing mitochondrial [Ca^2+^] ([Ca^2+^]_mt_). This indicated that local [Ca^2+^]_cyt_ can be very high in the proximity of IP_3_R channels, which is then sensed by the mitochondria, leading to mitochondrial Ca^2+^ accumulation. These findings were underpinned by perimitochondrial [Ca^2+^] measurements, showing that the local [Ca^2+^] was about 20-fold higher than global [Ca^2+^]_cyt_, allowing a “quasi-synaptic” transmission of the Ca^2+^ signal from the ER into the mitochondrial matrix (46). More precise determinations of local [Ca^2+^] at the ER–mitochondrial contact sites were obtained with pericam-tagged linkers, which indicated concentrations of ~10 µM (47). Importantly, mitochondrial Ca^2+^ transfer from the ER critically depended on IP_3_R-mediated Ca^2+^ release, since thapsigargin-induced depletion of the ER, which occurs *via* ER Ca^2+^-leak channels that are spread out over the ER membrane, was ineffective in eliciting a [Ca^2+^]_mt_ rise (46). Efficient IP_3_R-mediated Ca^2+^ transfer into the mitochondria is achieved by the molecular chaperone 75-kDa glucose-regulated protein (GRP75), which physically links IP_3_Rs to VDAC1 within the MAMs (32). Knockdown of GRP75 impairs the IP_3_R-mediated Ca^2+^ transfer to the mitochondria (32).

A positive feedback between the Ca^2+^ transfer from the ER to the mitochondria and the formation of H_2_O_2_ nanodomains at the ER–mitochondrial interface has recently been described (48). These H_2_O_2_ nanodomains are formed upon physiological stimulation of the IP_3_R-mediated Ca^2+^ transfer to the mitochondria. Ca^2+^ fuels the ETC, whose functionality determines the production of H_2_O_2_. In addition, Ca^2+^ accumulation in the matrix induced K^+^ flux, which results in drastically reduced volume of the cristae and subsequent H_2_O_2_ flux into the mitochondrial matrix, thereby increasing the mitochondrial matrix volume and squeezing out H_2_O_2_-rich fluid at the ER–mitochondrial interface. This locally released H_2_O_2_ sensitizes IP_3_Rs to low concentrations of agonists and stimulates Ca^2+^ oscillations, thereby further boosting mitochondrial bioenergetics.

### Translation of the Ca^2+^ Signals in the Mitochondria: Cell Survival, Autophagy, or Apoptosis

The adequate transfer of Ca^2+^ from the ER into the mitochondria requires a proper filling of the ER Ca^2+^ stores. Also, luminal Ca^2+^ controls IP_3_R-mediated Ca^2+^ release (49). Thus, lowering of the ER Ca^2+^ levels ([Ca^2+^]_ER_) will dampen ER–mitochondrial Ca^2+^ transfer, while increasing [Ca^2+^]_ER_ will augment ER–mitochondrial Ca^2+^ transfer. As a consequence, changes in the ER Ca^2+^-store content will eventually impact the level of Ca^2+^ transfer from the ER to the mitochondria and thus eventually cell death and survival decisions. A lowering of the ER Ca^2+^-store content has been shown to serve as a survival mechanism exploited by several pro-survival proteins and oncogenes, including Bax inhibitor-1 (BI-1), antiapoptotic Bcl-2 proteins, and Ras (50–53) as extensively described in Ref. (54). This does not only render cells more resistant to apoptotic triggers but it may also facilitate the survival of damaged or stressed cells, thereby resulting in oncogenesis and cancer cell survival. Several mechanisms can account for this [Ca^2+^]_ER_ lowering, including the function of BI-1 as an ER Ca^2+^-leak channel (55) and the sensitization of other ER Ca^2+^ channels that contribute to the ER Ca^2+^ leak, like IP_3_Rs (52, 56).

**Figure 1:**
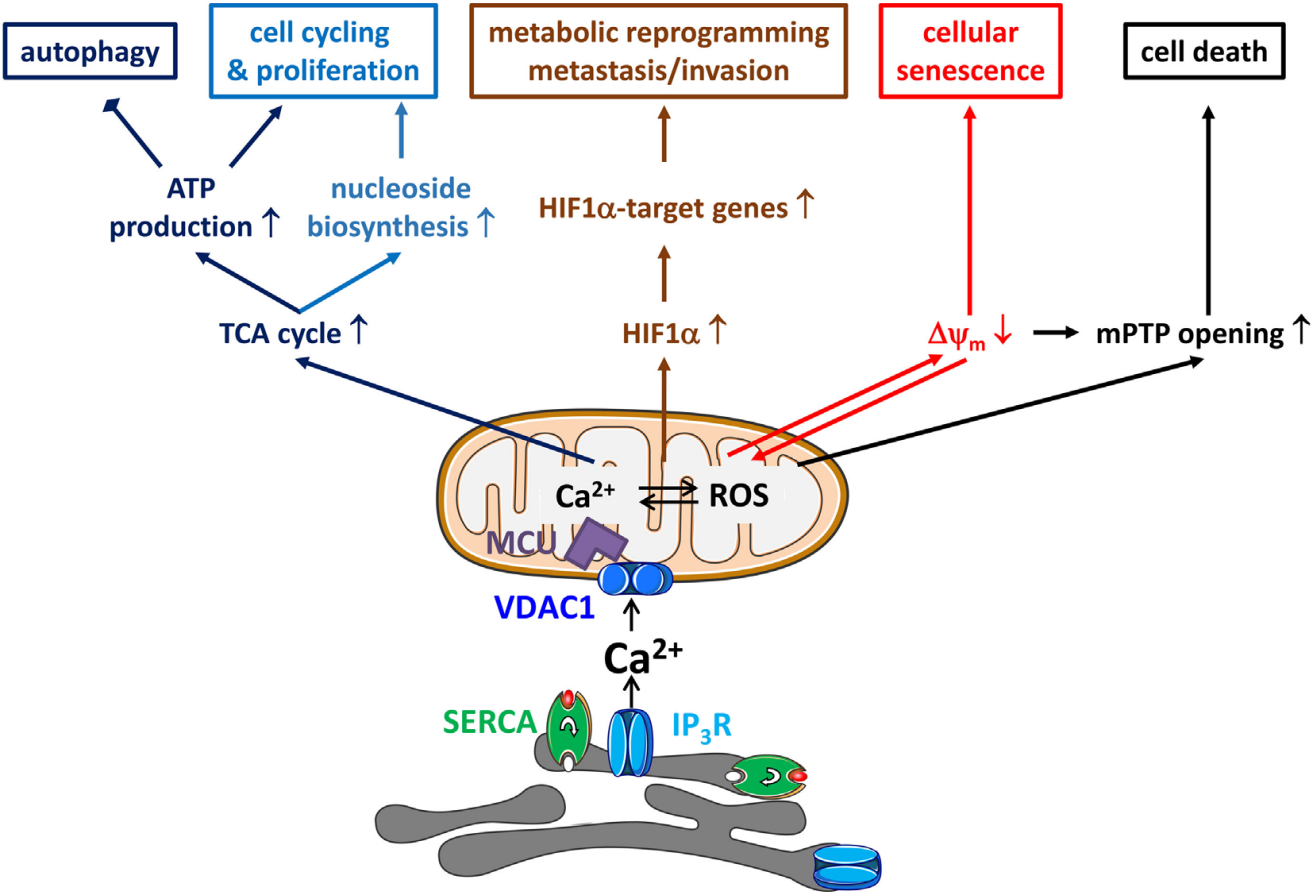
**Endoplasmic reticulum (ER)–mitochondrial Ca^2+^ transfers in cancer hallmarks**. ER–mitochondrial Ca^2+^ transfers will impact several hallmarks of cancer. First, ER-originating, inositol 1,4,5-trisphosphate receptor (IP_3_R)-driven Ca^2+^ signals delivered to the mitochondria will drive the tricarboxylic acid (TCA) cycle, which will not only result in ATP production *via* NADH and the electron transport chain but also in the production of mitochondrial substrates shuttled to biosynthetic pathways for macromolecules like nucleosides. This is accompanied by a decrease in autophagic flux due to a low activity of AMP-activated kinase. Second, mitochondrial Ca^2+^ signals will also increase mitochondrial reactive oxygen species (ROS) production, which will drive the transcription of the mitochondrial Ca^2+^ uniporter (MCU) regulates breast cancer progression *via* hypoxia-inducible factor 1α (HIF1α)—target genes with functions in metabolic reprogramming and, metastasis and invasion. Third, ER–mitochondrial Ca^2+^ fluxes are involved in mediating cellular senescence induced by oncogenes and replication. The mechanism involves the partial depolarization of the mitochondrial potential (Δψ_m_) and accumulation of ROS. Fourth, ER–mitochondrial Ca^2+^ fluxes impact cellular sensitivity toward apoptotic stimuli. In particular, mitochondrial Ca^2+^ overload, together with the accompanying ROS production, has been a critical factor for mitochondrial permeability transition pore (mPTP) opening. Thus, the cell death-inducing properties of several chemotherapeutics actually critically depend on their ability to elicit mitochondrial Ca^2+^ overload. Thus, ER–mitochondrial Ca^2+^ transfers display both oncogenic properties (cell cycling, proliferation, metabolic reprogramming, metastasis, and invasion) and tumor suppressive properties (reduced autophagy and increased cell death sensitivity).

The decrease in [Ca^2+^]_ER_ can either induce autophagy due to an impaired basal mitochondrial Ca^2+^ transfer (10) or suppress autophagy due to diminished [Ca^2+^]_cyt_ increases (57). On the one hand, low [Ca^2+^]_ER_ results in decreased spontaneous activity of IP_3_Rs, thereby abrogating its positive effect on the mitochondrial metabolism and resulting in the activation of AMP-activated kinase (AMPK) and subsequent increase in autophagic flux (10). Indeed, in many cells, IP_3_Rs appear to be constitutively active, thereby feeding Ca^2+^ in the mitochondria, which is necessary for mitochondrial metabolism (Figure 1). This is supported by observations made in DT40 B-lymphocytes in which all three IP_3_R isoforms have been deleted. These cells display a decreased mitochondrial NADH and ATP production due to a decreased activity of Ca^2+^-dependent dehydrogenases, the F1F0-ATPase, and the ETC (9, 58, 59). The decline in ATP levels results in the activation of AMPK, which inhibits the mammalian target of rapamycin (mTOR). In addition to mTOR suppression, AMPK promotes autophagy also through phosphorylation of unc-51-like kinase 1 (ULK1) and activation of the ULK1 complex (60, 61). However, the AMPK-dependent induction of autophagy upon inhibition of ER–mitochondrial Ca^2+^ transfer was shown to be mTOR-independent, suggesting a prominent role for the AMPK–ULK1 axis in this paradigm (10). Of note, while [Ca^2+^]_mt_ rises appear to suppress autophagy (62), [Ca^2+^]_cyt_ rises have been implicated in autophagy induction by the activation of calcium/calmodulin-dependent protein kinase kinaseβ (CaMKKβ), an upstream activator of AMPK. Thus, low [Ca^2+^]_ER_ can suppress autophagy by diminishing [Ca^2+^]_cyt_. It was proposed that antiapoptotic Bcl-2, by lowering the ER Ca^2+^ levels, could suppress cytosolic Ca^2+^ signals evoked by various pharmacological and physiological agents, thereby counteracting the activation of CaMKKβ-controlled autophagy (57, 63).

Further complexity of autophagy regulation arises from the fact that IP_3_R sensitization by accessory proteins might have an opposite outcome on autophagy, dependent on whether the sensitization is limited to MAMs or whether it occurs all over the ER membrane. Indeed, IP_3_R sensitization in the MAMs would lead to increased basal mitochondrial Ca^2+^ delivery, driving ATP production and thus suppressing autophagy. For example, Bcl-XL, which is present in the MAMs, can augment mitochondrial metabolism and is able to reduce autophagy by local IP_3_R sensitization in the MAMs (64, 65) (Table 1). In contrast, IP_3_R sensitization outside the MAMs will affect the overall ER Ca^2+^ loading due to an increased ER Ca^2+^ leak through IP_3_Rs that become sensitive to basal IP_3_ levels. This would result in partially depleted ER Ca^2+^ stores and decreased basal mitochondrial Ca^2+^ delivery, leading to reduced ATP production and increased autophagy. For example, BI-1, which presumably is ubiquitously present in the ER membrane, reduces the steady-state ER Ca^2+^ levels through IP_3_R sensitization, decreasing mitochondrial bioenergetics and thus inducing autophagy (66).

**Table 1:** **The impact of experimental, physiological, and cancer-related modulators of endoplasmic reticulum (ER)–mitochondrial Ca^2+^ flux on cell death, survival, and migration**.

Protein	Modulator	Mechanism	ER–mitochondrial Ca^2+^ flux	Cellular/*in vivo* effect	Model	Reference
IP_3_R	IP_3_R1/IP_3_R3 knockdown	IP_3_R1/IP_3_R3 expression ↓	↓	Cell death ↑ caused by mitotic catastrophe	HrasG12V-cyclin-dependent kinase 4 (CDK4) transformed human fibroblasts, tumorigenic cell lines: breast, prostate, and cervix	(67)
	XeB	Selective IP_3_R inhibitor		Autophagy ↑ as a cell survival mechanism	Primary fibroblasts; non-tumorigenic breast and prostate cell lines	
	U73122	PLC inhibitor (IP_3_↓)		Tumor size and weight ↓	B16F10 melanoma cell tumor xenograft (only performed with XeB)	
	IP_3_R2/IP_3_R3 knockdown	IP_3_R2/IP_3_R3 expression ↓	↓	Cell death ↑ caused by excessive autophagy	Breast cancer cell line (MCF-7)	(68)
	XeC^a^/2-APB^b^	Non-selective IP_3_R inhibitors		Tumor volume and weight ↓	Mouse 4T1 breast tumor model (only performed with 2-APB)	
	IP_3_R2 knockdown	IP_3_R2 expression ↓	↓	Escape from oncogene-induced senescence	Immortalized human mammary epithelial cells (HECs)	(69)
	BI-1	(Direct) sensitization of IP_3_Rs	↓	Autophagy ↑	HeLa and MEF cells	(56, 66)
		Functioning as an ER Ca^2^^+^-leak channel, mainly outside the mitochondria-associated ER membranes (MAMs)				(55)
	Bcl-XL	Direct sensitization of IP_3_Rs (at the MAMs), promoting pro-survival Ca^2+^ oscillations	↑	Cellular bioenergetics ↑	Reconstituted DT40-triple IP_3_R knockout cells	(64, 65, 70)
				Apoptosis ↓	CHO cells	
	PML	Counteracting Akt-mediated IP_3_R3 phosphorylation through PP2A recruitment	↑	Apoptosis ↑	MEF cells	(71, 72)
				Autophagy ↓	H1299	
					APL NB4 cells	
	Bcl-2	Direct inhibition of IP_3_Rs	↓	Apoptosis ↓	WEHI7.2 cells and Jurkat	(54, 73, 74)
	H_2_O_2_	Direct sensitization of IP_3_Rs (at the MAMs), *via* oxidation of specific thiol group of IP_3_R	↑	Cellular bioenergetics ↑	HEPG2	(48)

SERCA	TMX1	Binds and inhibits SERCA2b (at the MAMs) in a calnexin-dependent manner	↑	Apoptosis ↓	A375P melanoma and HeLa cell (xenograft)	(75, 76)
				Tumor growth ↓		
	p53 (extranuclear)	Accumulates at the ER and MAMs upon chemotherapy treatment, directly binding and activating SERCA2b by changing its oxidative state	↑	Apoptosis ↑	MEF, HeLa, and H1299 (human non-small cell lung carcinoma cell line)	(77, 78)
					HCT-116 and MDA-MB 468	
	Resveratrol	Reduced SERCA activity due to inhibition of mitochondrial ATP synthase	↑	Apoptosis ↑	Endothelial/epithelial cancer cell hybrid EA.hy926, HeLa	(79)

VDAC	Mcl-1	Binds and activates voltage-dependent anion channel type 1 (VDAC1) and VDAC3	↑	Cell migration ↑ caused by mitochondrial ROS ↑	NSCLC cell lines	(80)

MCU	MCU knockdown	MCU expression ↓	↓	Metastatic cell motility and matrix invasiveness ↓ caused by decreased mitochondrial ROS and HIF1-mediated transcription	Triple-negative breast cancers	(81)
					MDA-MB-231 xenografts	
				Cell death ↑ caused by mitotic catastrophe	HrasG12V-CDK4 transformed human fibroblasts; tumorigenic breast, prostate, and cervix (HeLa) cancer cell lines	(67)

	FATE1	Uncoupling of ER and mitochondria	↓	Apoptosis ↓	Adrenocortical carcinoma cells	(82)

*^a^Sarco/endoplasmic reticulum Ca^2+^-ATPase (SERCA) inhibitor*.

*^b^Store-operated Ca^2+^ entry and SERCA inhibitor*.

In contrast to the reduced mitochondrial Ca^2+^ supply, which triggers autophagy, it has become clear that excessive Ca^2+^ transfer from the ER to the mitochondria results in cell death (83–85) (Figure 1). This involves the opening of the mitochondrial permeability transition pore (mPTP) in the inner mitochondrial membrane, resulting in mitochondrial swelling and mitochondrial membrane rupture, eventually leading to cytochrome c release and apoptosis, if sufficient levels of ATP are available (85). Many cell death-inducing agents, like H_2_O_2_ (86, 87), arachidonic acid (88), ceramide (50, 86), and menadione (89, 90) have been shown to act at the ER by triggering Ca^2+^ release through IP_3_Rs and subsequently provoking mitochondrial Ca^2+^ rises (91). Moreover, the ability of chemotherapeutics, like adriamycin (77), arsenic trioxide (71), and mitotane (82) and of photodynamic therapy (78) to kill cancer cells strongly depends on their ability to adequately induce ER–mitochondrial Ca^2+^ transfer (92). The spectrum of chemotherapeutics acting in this way might be quite broad, since recently it was shown that cisplatin and topotecan increase [Ca^2+^]_cyt_ over time, although [Ca^2+^]_mt_ was not determined (93). The transfer of pro-apoptotic Ca^2+^ signals to the mitochondria appears to be mediated by VDAC1 and not VDAC2 or VDAC3 (86). Also further insights in the mechanism underlying mPTP opening upon mitochondrial Ca^2+^ overload have been obtained. Ca^2+^ accumulating in the mitochondrial matrix binds to cardiolipin, which dissociates from the respiratory chain complex II and eventually results in its disassembly. The unleashed subunits of complex II produce reactive oxygen species (ROS) in the mitochondrial matrix, resulting in the opening of the mPTP (94).

The dichotomous impact of mitochondrial Ca^2+^ on both apoptosis and autophagy implies that reduced mitochondrial Ca^2+^ transfer will simultaneously result in acquired resistance to apoptotic stimuli and in increased autophagy (Figure 1) (95). This mechanism has been shown to be responsible for the sustained proliferation of cells deficient in promyelocytic leukemia protein (PML), a tumor suppressor present at the MAMs that augments ER–mitochondrial Ca^2+^ flux on the one hand and excessive chemotherapeutic resistance on the other hand (71, 72). Indeed, loss of PML reduced basal ER–mitochondrial Ca^2+^ transfers, thereby inducing sustained autophagy, promoting malignant cell survival and reduced chemotherapy-induced apoptosis contributing to poor chemotherapeutic efficacy (Table 1).

Finally, it is important to remark that cell death and survival are regulated by mitochondrial dynamics, including mitochondrial fusion, mainly mediated by optic atrophy 1 and by dynamin-related GTPases mitofusin-1 (Mfn-1) and Mfn-2, and mitochondrial fission, mainly mediated by the cytosolic soluble dynamin-related protein 1 (Drp1) (96, 97). Mitochondrial fragmentation leads to Bax-dependent apoptosis, while hyperfusion of mitochondria in response to a decline in Drp1 results in proliferation. Moreover, mitochondrial dynamics themselves are also regulated by Ca^2+^ signaling *via* calcineurin-mediated dephosphorylation of Drp1 (98). Mitochondrial hyperfusion may also render cells more sensitive to apoptotic stimuli due to hyperpolarization of the mitochondrial membrane and thus an increased driving force for mitochondrial Ca^2+^ uptake (99, 100). Hyperpolarization of mitochondrial membrane is also tightly connected to ROS production and release. As such, extensive ROS generation results in hyperpolarization of the mitochondrial membrane, followed by amplified ROS generation. ROS are released into the cytosol, where they can affect other mitochondria. This process is called ROS-induced ROS release and it could play important role in mitochondrial and cellular injuries (101).

## Cancer Cells’ Addiction to Constitutive ER–Mitochondrial Ca^2+^ Signaling

Clearly, basal IP_3_R-driven Ca^2+^ signals and subsequent ER–mitochondrial Ca^2+^ transfer impact cell death and survival processes. Inhibition of IP_3_Rs and thus spontaneous Ca^2+^ signals lead to reduced mitochondrial bioenergetics and increased autophagy, allowing cell survival (10). Recently, the role of basal IP_3_R-mediated Ca^2+^ signaling and ER–mitochondrial Ca^2+^ transfer for cancer cell survival was investigated in more detail (67). A comparison was made between non-tumorigenic and tumorigenic cell lines, as well as between non-transformed primary human fibroblasts and fibroblasts transformed by the ectopic expression of oncogenic HRasG12V and cyclin-dependent kinase 4. For reasons of clarity, we will refer to the former as “normal cells” and to the latter as “cancer cells.” Strikingly, inhibition of IP_3_R activity, knockdown of IP_3_R or MCU led in both normal and cancer cells to a so-called “bioenergetic crisis” characterized by a decreased basal and maximal oxygen consumption rate and increased AMPK phosphorylation, subsequently resulting in an increased autophagic flux. However, these interventions resulted in cell death in the cancer cells but not in the normal cells, indicating that autophagy upregulation induced upon IP_3_R inhibition was sufficient to sustain cell survival in the normal cells but not in cancer cells (67). Similar results were obtained in another recent study, which also implicated autophagy induced by IP_3_R inhibition in cancer cell death (68). Selective knockdown of IP_3_R isoforms using siRNA or general IP_3_R inhibition using 2-APB or xestospongin C compromised mitochondrial bioenergetics, led to generation of ROS, activation of AMPK, and upregulation of Atg5, an essential autophagy gene. This resulted in excessive autophagy in the cancer cells. Cells could be rescued by ROS scavengers and autophagy inhibitors, indicating that autophagy was at least in part responsible for the cell death. 2-APB was also used in xenograft models, where it strongly suppressed *in vivo* tumor growth. It is important to note that 2-APB and xestospongin C cannot be considered as selective inhibitors of IP_3_Rs and thus their impact on cancer cell survival might be related to off-target effects (Table 1).

However, the fact that in some conditions autophagy upregulation is not sufficient for cancer cell survival upon IP_3_R inhibition is in striking contrast to the important role of autophagy for cancer cell survival in conditions of nutrient starvation (102). Ras-driven lung cancer cells were dependent on autophagy for their survival during starvation conditions. Consistent with this, caloric restriction was more effective to suppress Ras-driven tumor growth when it was combined with autophagy inhibition (103). This may indicate that the contribution of autophagy for cancer cell survival might be different dependent on the way autophagy was induced (IP_3_R inhibition *versus* starvation), which may be due to differences in the produced breakdown products and their usage in metabolic and biosynthetic pathways (104). The cancer cell death induced by IP_3_R inhibition could be rescued by providing the cells with cell-permeable mitochondrial substrates like methyl pyruvate, that is oxidized to NADH necessary to drive OXPHOS and production of ATP or dimethyl α-ketoglutarate, a precursor for glutamine to fuel the TCA cycle, where it enters and is oxidized by a Ca^2+^-dependent α-KGDH as the first step (67). Moreover, the protective effects of the substrates in xestospongin B-treated cancer cells were unrelated to their antioxidant properties, since the antioxidant *N*-acetyl-cysteine could not protect the cancer cells against cell death. Instead, nucleoside complementation could rescue the death of the cancer cells induced by IP_3_R inhibition (67), indicating that constitutive ER–mitochondrial Ca^2+^ fluxes are required for cancer cell survival by sustaining an adequate source of mitochondrial substrates for nucleotide synthesis. This phenomenon was also observed *in vivo*, where tumor growth could be reduced upon treatment with xestospongin B.

**Figure 2:**
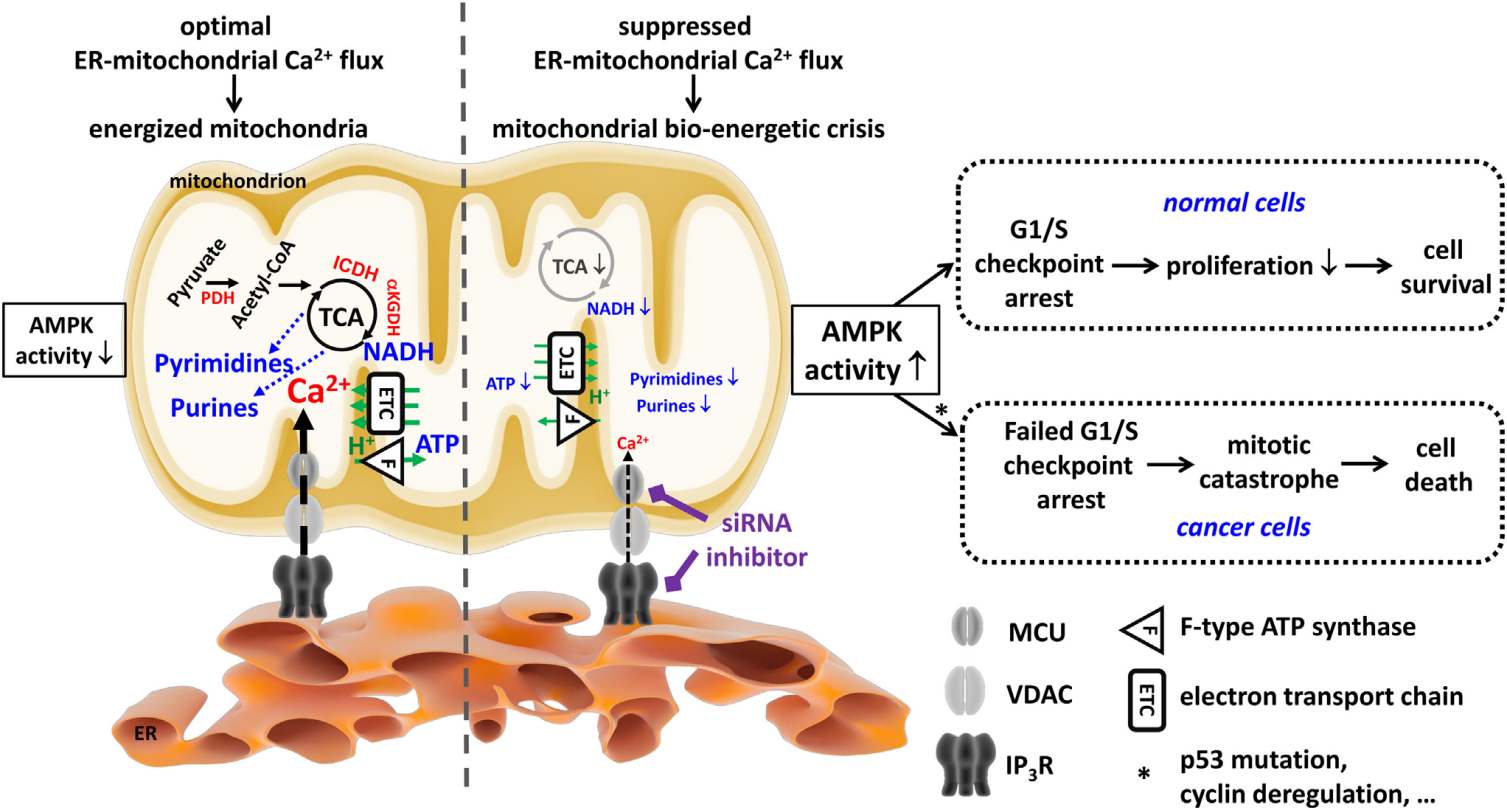
**Cancer cells are addicted to endoplasmic reticulum (ER)–mitochondrial Ca^2+^ fluxes to produce tricarboxylic acid (TCA)-dependent mitochondrial substrates used to sustain their uncontrolled proliferation**. In both non-malignant and malignant cells, mitochondria require Ca^2+^ from the ER Ca^2+^ store for an adequate performance of the TCA cycle, which ultimately leads to energy production (ATP), redox homeostasis (NADH), and anabolism, e.g., of pyrimidine and purine nucleotides. The Ca^2+^-dependent control of the TCA cycle is due to the Ca^2+^-dependent activity of several rate-limiting enzymes (PDH, ICDH, and αKGDH, all indicated in red). Ca^2+^ is efficiently delivered to the mitochondria in a quasi-synaptic manner involving Ca^2+^-signaling microdomains established at mitochondria-associated ER membranes involving inositol 1,4,5-trisphosphate receptor (IP_3_R), voltage-dependent anion channel type 1 (VDAC1), and mitochondrial Ca^2+^ uniporter (MCU) as Ca^2+^-transport systems. Of note, although cancer cells switch to glycolysis for their ATP production, they too rely on functional mitochondria for the production of mitochondrial substrates used for anabolic processes, like the generation of nucleotides required for the DNA synthesis necessary for their deregulated cell cycle progression and proliferation. Ablation of these ER–mitochondrial Ca^2+^ fluxes (e.g., by using siRNA-based approaches or pharmacological inhibitors like xestospongin B) results in compromised mitochondrial bioenergetics, causing a decline in ATP, NADH, and nucleotides. In both non-malignant and malignant cells, this leads to an increase in AMP-activated kinase (AMPK) activity. However, in non-malignant cells, increased AMPK activity will result in an arrest at the G1/S checkpoint, likely involving p53 activation and cyclin E downregulation, which will dampen proliferation as a cell survival strategy. In malignant cells, the link between AMPK activity and the G1/S checkpoint is lost (e.g., due to p53 mutations or cyclin deregulation). As a consequence, despite the mitochondrial bioenergetic crisis and the lack of mitochondrial substrates for DNA synthesis, cancer cells will progress toward the S phase and mitosis. This results in necrotic cell death due to mitotic catastrophe. This figure was originally published in Ref. (105). © 2016 Geert Bultynck. A copyright license to republish this figure has been obtained.

Indeed, in conditions of suppressed ER–mitochondrial Ca^2+^ flux, normal cells display slower cell-cycle progression and become arrested at the G1/S checkpoint. This prevents DNA synthesis and shifts cells to accumulate in the G1 phase rather than the S phase (Figure 2), ultimately reducing the rate of daughter cell generation and proliferation. Conversely, cancer cells exposed to IP_3_R inhibitors have lost proper control over their G1/S checkpoint, progressing through the cell cycle and undergoing mitosis irrespective of their OXPHOS and mitochondrial bioenergetic status. As such, cancer cells will divide even though their mitochondrial metabolism is insufficient to cope with the anabolic pathways needed to make a living daughter cell, eventually resulting in a “mitotic catastrophe” upon daughter cell separation (67). Interestingly, arresting cancer cells in the G1/S phase and preventing them to undergo mitosis strongly suppressed cell death induced by IP_3_R inhibition. Hence, beyond the well-established roles of IP_3_Rs in apoptosis, these data reveal that, in the absence of proper cell-cycle control, cells are addicted to constitutive IP_3_R function and sustained ER–mitochondrial Ca^2+^ transfer for fueling mitochondrial metabolism. These ER–mitochondrial Ca^2+^ fluxes maintain sufficiently high levels of TCA cycling by ensuring the activity of Ca^2+^-dependent dehydrogenases, thereby delivering an adequate supply of mitochondrial substrates required for nucleotide production and DNA synthesis during ongoing proliferation (67).

Interestingly, the need for adequate mitochondrial Ca^2+^ signaling in tumor cells is further supported by a very recent study performed in triple-negative breast cancer (81). It was shown that MCU expression positively correlated with the metastatic phenotype and clinical stage of the breast cancers, while the expression of MCUb, a negative regulator of MCU (37), displayed a negative correlation. Strikingly, silencing of MCU blunted cell invasiveness without affecting cell viability. The *in vivo* growth of breast cancer cells in which MCU was deleted was severely impaired, correlating with an altered cellular redox state and impaired mitochondrial production of ATP. In this mechanism, MCU-mediated Ca^2+^ uptake in the mitochondria resulted in increased ROS production and activation of hypoxia-inducible factor 1α signaling, contributing to tumor growth and metastatic behavior (81). Similar results were reported in NSCLS cells where mitochondrial ROS generation and increased cell migration were correlating to enhanced [Ca^2+^]_mt_ uptake through Mcl-1/VDAC interaction (80) (Table 1).

**Figure 3:**
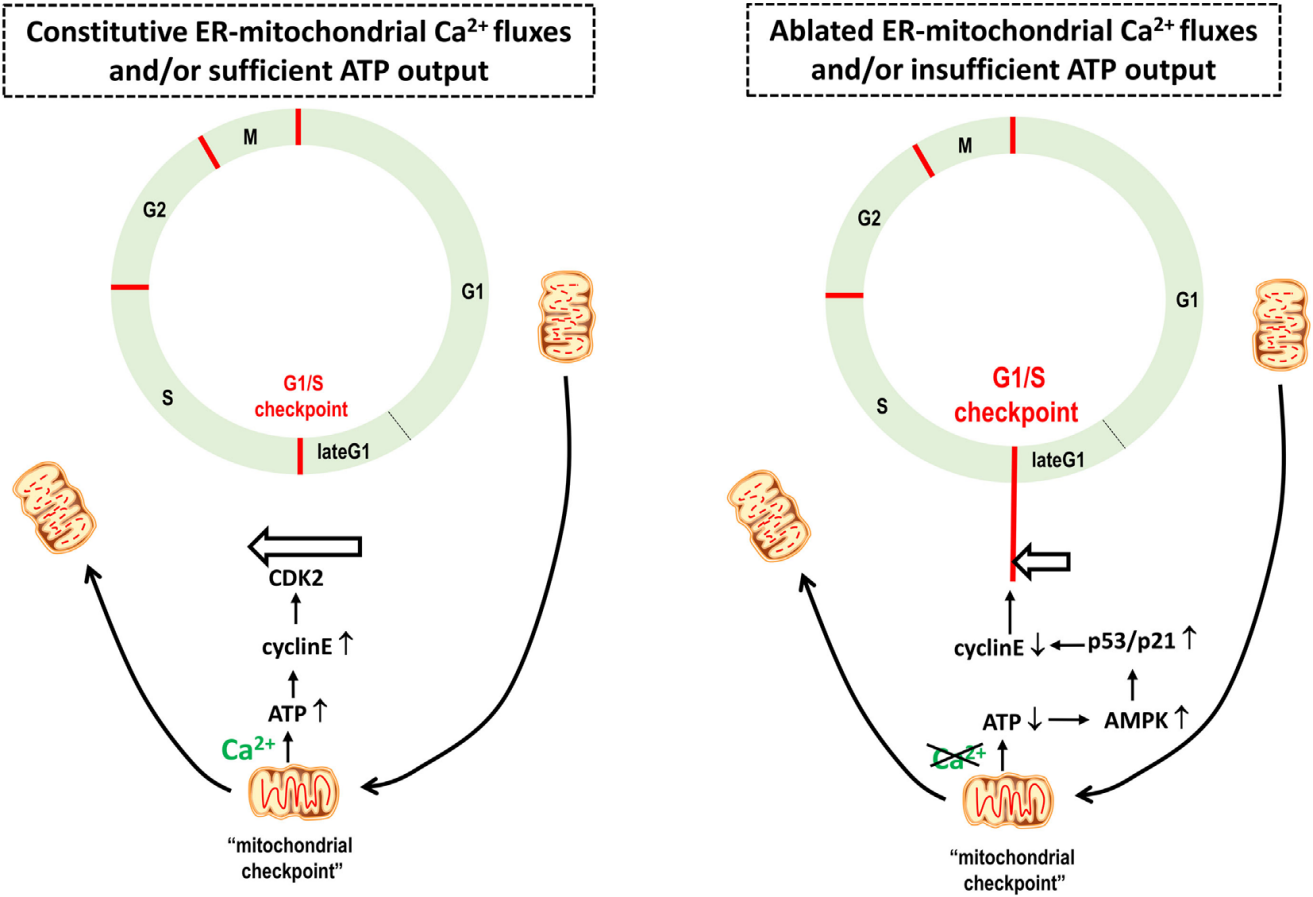
**A boost in mitochondrial ATP output provides a mitochondrial checkpoint for cellular health, enabling cells to bypass the G1/S checkpoint and cell cycle progression**. Based on Mitra et al. (106), mitochondrial structure changes along the cell cycle progression. At the late G1 phase, the mitochondrial structure changes into a giant, single tubular network, electrically coupled and hyperpolarized, boosting ATP production. The G1–S transition that ensures the initiation of DNA replication is controlled by the cyclin E, which, in turn, further binds and activates CDK2. Cyclin E is upregulated upon increased ATP output, enabling S-phase progression and proliferation. Non-tumorigenic cells experiencing a reduction of ATP production due to compromised mitochondrial bioenergetics will trigger the G1/S checkpoint arrest due to AMP-activated kinase (AMPK) activation and subsequent phosphorylation and activation of the tumor suppressor protein p53 that in turn downregulates cyclin E protein levels. In tumorigenic cells, it is anticipated that this tight link between adequate mitochondrial bioenergetics and the G1/S checkpoint is lost. Hence, cancer cells can progress through the cell cycle irrespective of their mitochondrial bioenergetic status. Thus, a mitochondrial bioenergetic crisis will slow down the cell cycle and proliferation in normal cells, while in cancer cells, the cell cycle will continue, eventually resulting in a mitotic catastrophe.

Further studies are necessary in order to determine how cancer cells escape from the G1/S checkpoint with impaired mitochondrial bioenergetics due to reduced ER–mitochondrial Ca^2+^ fluxes. However, an important link between mitochondrial dynamics and cell-cycle control was described (106). This study revealed that at the G1/S checkpoint the mitochondrial structure changes into a single tubular network, electrically coupled and hyperpolarized, boosting ATP production (Figure 3) (106). The progression of the cell cycle is ensured by specific cyclins associated with CDKs (107). The G1-to-S transition, which ensures the initiation of DNA replication, is controlled by cyclin E, which, in turn, further binds and activates CDK2 to phosphorylate downstream targets for DNA production. Cyclin E abundance is restricted to the transition from the G1 phase to the S phase and decreases with the progression of the cell cycle. Mitochondrial hyperfusion will support ATP production and as such cyclin E stability, enabling S-phase progression. This actually establishes an important “mitochondrial checkpoint” that only permits G1/S progression when mitochondrial bioenergetics and cellular health are adequate (Figure 2). Based on the model proposed by Finkel and Hwang (108), cells with impaired mitochondrial bioenergetics and, thus reduced ATP output and increased AMPK activity, will activate p53 and p21, a cell cycle regulator, leading to a drop in cyclin E (109) and an arrest of the cells at the G1 phase due to their inability to overcome the G1/S checkpoint (Figure 3). In light of the requirement for a burst of ATP production for proper S phase progression, an increased mitochondrial Ca^2+^ demand would also be expected. Therefore, further work is required to establish whether IP_3_R activity and ER–mitochondrial tethering and/or Ca^2+^ transfers could become enhanced at the G1/S transition to support this increased ATP production as part of the “mitochondrial checkpoint.” Previous studies have implicated IP_3_R sensitization as a critical step during G1/S transition and identified IP_3_Rs as targets for cyclins and substrates for CDKs (110). However, in cancer cells, the G1/S checkpoint control appears to be lost despite the fact that IP_3_R inhibition still leads to activation of AMPK, implying defects in the mechanisms linking AMPK to the G1/S checkpoint arrest, for example, mutations impairing p53 activity or hyperactivating CDKs. Previous work indicated that p53 mutations could result in a bypass of G1/S arrest (108). Thus, re-expression of p53 may restore the G1/S checkpoint control in a number of these cancer cell types exposed to IP_3_R inhibition, thereby slowing down cell cycle progression and proliferation and preventing cell death by mitotic catastrophe.

In addition to the addiction of some cancer cells to constitutive ER–mitochondrial Ca^2+^ fluxes, ER–mitochondrial contact sites and Ca^2+^-signaling events might be altered to favor cancer cell survival. This concept is supported by another recent study. TMX1, a redox-sensitive oxidoreductase that is enriched in the MAMs in a palmitoylation-dependent manner, was shown to regulate mitochondrial bioenergetics and *in vivo* tumor growth by controlling ER–mitochondrial Ca^2+^ signaling (75, 76). Upon palmitoylation, TMX1 is recruited to the MAMs, where it binds and inhibits SERCA2b. As such, loss of TMX1 accelerates SERCA2b-mediated ER Ca^2+^ accumulation, particularly in the MAMs. As a consequence, loss of TMX1 in HeLa and A375P, a malignant melanoma cell line, increased ER Ca^2+^ retention and reduced ER–mitochondrial Ca^2+^ transfer. This led to a reduction in mitochondrial bioenergetics, thereby lowering ATP production and the oxygen consumption rate. Consistent with the work of Foskett and others (67), loss of TMX1 resulted in increased cell death and increased ROS production *in vitro* (Table 1). However, *in vivo*, opposite findings were obtained. Furthermore, while loss of TMX1 in these cancer cell lines accelerated tumor growth, TMX1 overexpression had the opposite effect (75). This might be due to the contribution of the microenvironment, including reduced accessibility of oxygen and nutrients, which may contribute to mitochondrial stress. Interestingly, it was shown that although cancer cells lacking TMX1 proliferate slower and display more spontaneous cell death, they are more resistant to mitochondrial stress inducers like rotenone and antimycin (75). Hence, *in vivo*, cancer cells may experience ongoing mitochondrial stress and/or shortage of nutrients. Under such conditions, cancer cells that have lost TMX1 expression might have a growth advantage over cancer cells with high TMX1 expression. Alternatively, these cells may display increased autophagy, which is beneficial for cancer cell survival under starvation conditions by providing mitochondrial substrates that feed the TCA cycle and sustain nucleotide biosynthesis (102, 104). However, further work is needed to understand these aspects in more detail. In particular, the differences between IP_3_R inhibition and loss of TMX1,which both impair mitochondrial bioenergetics and result in spontaneous cell death *in vitro* but lead to an opposite effect in *in vivo* tumor growth experiments (impaired upon IP_3_R inhibition *versus* accelerated upon TMX1 loss) require further research.

## ER–Mitochondrial Ca^2+^ Signaling Underlying Cellular Senescence and Cancer Cell Death Therapies

It is important to note that alterations in ER–mitochondrial Ca^2+^ transfers will not only impact mitochondrial bioenergetics but also cancer cell senescence and sensitivity toward chemotherapeutic drugs (Figure 1).

Adequate ER–mitochondrial Ca^2+^ transfer has been implicated in oncogene-induced and replicative senescence, a condition characterized by a stable proliferation arrest (69, 111). Cancer cells in which IP_3_R2, the most sensitive isoform to its ligand IP_3_, or MCU were knocked down could escape cellular senescence (69). Conversely, cancer cells exposed to a continuous supply of cell-permeable IP_3_ displayed premature senescence. Strikingly, cells undergoing oncogene-induced senescence displayed an increase in basal mitochondrial Ca^2+^ and IP_3_-induced mitochondrial Ca^2+^ accumulation. Cells lacking IP_3_R2 or MCU did not display this mitochondrial Ca^2+^ rise. Mitochondrial Ca^2+^ induced cellular senescence by causing a partial depolarization of the mitochondrial membrane and an accumulation of mitochondrial ROS. Moreover, cellular senescence could be mimicked by mitochondrial depolarization by the mitochondrial uncoupler FCCP (69). A further detailed discussion on the alterations in mitochondrial homeostasis and the contributing underlying mechanisms in cellular senescence is provided elsewhere (112).

The adequate ER–mitochondrial Ca^2+^ transfer underlies the cell death-inducing properties of several chemotherapeutic drugs. Recently, extranuclear p53 has emerged as an important molecular link between chemotherapeutic responses and Ca^2+^ signaling (77, 113). Upon exposure to chemotherapeutic drugs, p53 was shown to accumulate at the ER membranes where it increases SERCA2b activity (Table 1). This resulted in increased [Ca^2+^]_ER_, increasing the likelihood of pro-apoptotic Ca^2+^ transfers to the mitochondria. Cells that lack p53 or that express oncogenic p53 mutations failed to upregulate SERCA2b activity and display ER–mitochondrial Ca^2+^ transfers and cell death (72). In addition, cells that lack p53 can be sensitized to chemotherapy by overexpressing SERCA or MCU, facilitating ER–mitochondrial Ca^2+^ transfer (78). Thus, downregulation of ER–mitochondrial Ca^2+^ fluxes may not only favor cancer cell survival (e.g., by upregulating autophagy) but could also lead to cell-death resistance, as has been shown recently for tumor cells lacking PML (71) or FATE1 (82). FATE1 is a cancer-testis antigen, which localizes at the ER–mitochondrial interface (82). Recently, it has been identified as an MAMs spacer, thereby impairing mitochondrial Ca^2+^ uptake. As a consequence, FATE1 upregulation, like in adrenocortical carcinoma cells, results in cell-death resistance not only in response to pro-apoptotic stimuli that impinge on ER–mitochondrial Ca^2+^ signaling but also in response to mitotane, a chemotherapeutic drug clinically used in the treatment of patients with adrenocortical cancer. Moreover, FATE1 expression is also inversely correlated with the overall survival of adrenocortical cancer patients (82). Oppositely, enhancing ER–mitochondrial Ca^2+^ transfer will favor cell-death therapies (92). Interestingly, some anticancer drugs might actually impact ER–mitochondrial contact sites and thereby enhance the response to other chemotherapeutics. For instance, ABT-737, a non-selective Bcl-2/Bcl-XL inhibitor (114, 115) could reverse the cisplatin resistance in ovarian cancer cells due to increased ER–mitochondrial Ca^2+^ contact sites (116). Specifically, the authors demonstrated that ABT-737 enriched cisplatin-induced GRP75 and Mfn-2 content at the ER–mitochondria interface. The latter event led to enhanced mitochondrial Ca^2+^ overload and subsequent cell death (116). Moreover, tumor suppressors at MAMs, including p53, were reported to modulate Ca^2+^ transfer and the contact sites (54). Another anticancer compound, whose mechanism involves a Ca^2+^-dependent step is resveratrol (79). This natural compound selectively increased the mitochondrial Ca^2+^ uptake of cancer cells, while normal cells remained unaffected. Similarly to other phenols, resveratrol inhibits ATP synthase and impairs ATP production, thereby decreasing mitochondrial [ATP] without affecting cytosolic [ATP] (117). This resulted in suppressed SERCA activity, particularly at the MAM interface, thereby increasing the net flux of Ca^2+^ through IP_3_Rs and augmenting mitochondrial uptake (Table 1). The striking difference between the mitochondrial Ca^2+^ uptake in cancer and in normal cells in the presence of resveratrol was attributed to the enhanced and more stable MAMs in cancer cells, which facilitate the ER–mitochondrial Ca^2+^ transfer (79). In addition to this, resveratrol can induce autophagy *via* a mechanism that requires cytosolic Ca^2+^ and the presence of IP_3_Rs. In this study, resveratrol triggered a depletion of the ER in intact cells independently of IP_3_Rs, but not in permeabilized cells where Ca^2+^ stores are loaded by application of ATP, arguing against a direct inhibition of SERCA by resveratrol. Thus, these findings may relate to an *in cellulo* decline in SERCA activity due to a decline in ATP (118).

## Conclusion

Endoplasmic reticulum–mitochondrial Ca^2+^ fluxes impact several cancer hallmarks, including mitochondrial metabolism, autophagy, apoptosis resistance, and metastasis. It is very likely that different tumor stages require different levels of ER–mitochondrial Ca^2+^ flux for instance to ensure cell survival at early stages, promote invasion at intermediate stages and tumor growth at late stages. Moreover, different oncogenes and tumor suppressors exert their part of their function at the MAMs by impacting Ca^2+^-transport systems.

An emerging concept is that cancer cells become addicted to constitutive ER–mitochondrial Ca^2+^ transfers. Thus, suppressing these basal ongoing ER–mitochondrial Ca^2+^ fluxes represent a therapeutic strategy to target tumor cells thereby suppressing their survival, invasion and growth.

In contrast to this, ER–mitochondrial Ca^2+^ fluxes appear instrumental for proper therapeutic responses to chemotherapeutic drugs, since an adequate ER–mitochondrial Ca^2+^ transfer is important for their cell death-inducing properties. Hence, enhancing ER–mitochondrial Ca^2+^ transfer may provide an attractive strategy to overcome cell death resistance of certain types of cancer toward chemotherapeutics.

Hence, it is expected that both dampening and boosting ER–mitochondrial Ca^2+^ transfers hold therapeutic potential, dependent on the clinical stage of the tumor and the applied anticancer strategy. However, a major challenge will be to limit these effects to cancer cells, as obviously ER–mitochondrial Ca^2+^ fluxes also underlie the survival of healthy cells. Nevertheless, the presence, composition, and properties of ER–mitochondrial contact sites in healthy *versus* cancer cells and the dependence of these cells on these sites for cell survival may be strikingly different, creating a therapeutic window for the selective targeting of cancer cells while sparing healthy cells.

## Author Contributions

GB and RR drafted the manuscript. HI, MK, RR, and GB wrote parts of the manuscripts. HI, RR, and GB prepared figures. All the authors have read and approved the final version of the manuscript.

## Conflict of Interest Statement

The authors declare that the research was conducted in the absence of any commercial or financial relationships that could be construed as a potential conflict of interest. The reviewer, IS, and handling editor declared their shared affiliation, and the handling editor states that the process nevertheless met the standards of a fair and objective review.

## References

[1] Greiner EF Guppy M Brand K. Glucose is essential for proliferation and the glycolytic enzyme induction that provokes a transition to glycolytic energy production. J Biol Chem (1994) 269:31484–90. 7989314

[2] Lunt SY Vander Heiden MG. Aerobic glycolysis: meeting the metabolic requirements of cell proliferation. Annu Rev Cell Dev Biol (2011) 27:441–64. 10.1146/annurev-cellbio-092910-154237 21985671

[3] Warburg O Wind F Negelein E The metabolism of tumors in the body. J Gen Physiol (1927) 8:519–30. 10.1085/jgp.8.6.519 19872213 PMC2140820

[4] Olson KA Schell JC Rutter J. Pyruvate and metabolic flexibility: illuminating a path toward selective cancer therapies. Trends Biochem Sci (2016) 41:219–30. 10.1016/j.tibs.2016.01.002 26873641 PMC4783264

[5] Liberti MV Locasale JW. The Warburg effect: how does it benefit cancer cells? Trends Biochem Sci (2016) 41:211–8. 10.1016/j.tibs.2015.12.001 26778478 PMC4783224

[6] Pfeiffer T Schuster S Bonhoeffer S. Cooperation and competition in the evolution of ATP-producing pathways. Science (2001) 292:504–7. 10.1126/science.1058079 11283355

[7] Boroughs LK DeBerardinis RJ. Metabolic pathways promoting cancer cell survival and growth. Nat Cell Biol (2015) 17:351–9. 10.1038/ncb3124 25774832 PMC4939711

[8] Desler C Lykke A Rasmussen LJ The effect of mitochondrial dysfunction on cytosolic nucleotide metabolism. J Nucleic Acids (2010) 2010:701518 10.4061/2010/701518 20862377 PMC2938461

[9] Rizzuto R De Stefani D Raffaello A Mammucari C. Mitochondria as sensors and regulators of calcium signalling. Nat Rev Mol Cell Biol (2012) 13:566–78. 10.1038/nrm3412 22850819

[10] Cárdenas C Miller RA Smith I Bui T Molgó J Müller M Essential regulation of cell bioenergetics by constitutive InsP_3_ receptor Ca^2+^ transfer to mitochondria. Cell (2010) 142:270–83. 10.1016/j.cell.2010.06.007 20655468 PMC2911450

[11] MacLennan DH Rice WJ Green NM The mechanism of Ca^2+^ transport by sarco(endo)plasmic reticulum Ca^2+^-ATPases. J Biol Chem (1997) 272:28815–8. 10.1074/jbc.272.46.28815 9360942

[12] Michalak M Robert Parker JM Opas M. Ca^2+^ signaling and calcium binding chaperones of the endoplasmic reticulum. Cell Calcium (2002) 32:269–78. 10.1016/S0143416002001884 12543089

[13] Mikoshiba K. Role of IP_3_ receptor signaling in cell functions and diseases. Adv Biol Regul (2015) 57:217–27. 10.1016/j.jbior.2014.10.001 25497594

[14] Berridge MJ. Inositol trisphosphate and calcium signalling mechanisms. Biochim Biophys Acta (2009) 1793:933–40. 10.1016/j.bbamcr.2008.10.005 19010359

[15] Jaimovich E Mattei C Liberona JL Cardenas C Estrada M Barbier J Xestospongin B, a competitive inhibitor of IP_3_-mediated Ca^2+^ signalling in cultured rat myotubes, isolated myonuclei, and neuroblastoma (NG108-15) cells. FEBS Lett (2005) 579:2051–7. 10.1016/j.febslet.2005.02.053 15811317

[16] MacMillan D McCarron J The phospholipase C inhibitor U-73122 inhibits Ca^2+^ release from the intracellular sarcoplasmic reticulum Ca^2+^ store by inhibiting Ca^2+^ pumps in smooth muscle: U-73122 inhibits intracellular Ca^2+^ release. Br J Pharmacol (2010) 160:1295–301. 10.1111/j.1476-5381.2010.00771.x 20590621 PMC2938802

[17] Maruyama T Kanaji T Nakade S Kanno T Mikoshiba K. 2APB, 2-aminoethoxydiphenyl borate, a membrane-penetrable modulator of Ins(1,4,5)P3-induced Ca^2+^ release. J Biochem (1997) 122:498–505. 10.1093/oxfordjournals.jbchem.a021780 9348075

[18] Gafni J Munsch JA Lam TH Catlin MC Costa LG Molinski TF Xestospongins: potent membrane permeable blockers of the inositol 1,4,5-trisphosphate receptor. Neuron (1997) 19:723–33. 10.1016/S0896-6273(00)80384-0 9331361

[19] DeHaven WI Smyth JT Boyles RR Bird GS Putney JW. Complex actions of 2-aminoethyldiphenyl borate on store-operated calcium entry. J Biol Chem (2008) 283:19265–73. 10.1074/jbc.M801535200 18487204 PMC2443677

[20] Missiaen L Callewaert G De Smedt H Parys JB. 2-Aminoethoxydiphenyl borate affects the inositol 1,4,5-trisphosphate receptor, the intracellular Ca^2+^ pump and the non-specific Ca^2+^ leak from the non-mitochondrial Ca^2+^ stores in permeabilized A7r5 cells. Cell Calcium (2001) 29:111–6. 10.1054/ceca.2000.0163 11162848

[21] Bittremieux M Gerasimenko JV Schuermans M Luyten T Stapleton E Alzayady KJ DPB162-AE, an inhibitor of store-operated Ca^2+^ entry, can deplete the endoplasmic reticulum Ca^2+^ store. Cell Calcium (2017) 62:60–70. 10.1016/j.ceca.2017.01.015 28196740

[22] Leon-Aparicio D Pacheco J Chavez-Reyes J Galindo JM Valdes J Vaca L Orai3 channel is the 2-APB-induced endoplasmic reticulum calcium leak. Cell Calcium (2017). 10.1016/j.ceca.2017.01.012 28179072

[23] Leon-Aparicio D Chavez-Reyes J Guerrero-Hernandez A. Activation of endoplasmic reticulum calcium leak by 2-APB depends on the luminal calcium concentration. Cell Calcium (2017). 10.1016/j.ceca.2017.01.013 28249687

[24] De Smet P Parys JB Callewaert G Weidema AF Hill E De Smedt H Xestospongin C is an equally potent inhibitor of the inositol 1,4,5-trisphosphate receptor and the endoplasmic-reticulum Ca^2+^ pumps. Cell Calcium (1999) 26:9–13. 10.1054/ceca.1999.0047 10892566

[25] Fill M Copello JA. Ryanodine receptor calcium release channels. Physiol Rev (2002) 82:893–922. 10.1152/physrev.00013.2002 12270947

[26] Protasi F Structural interaction between RYRs and DHPRs in calcium release units of cardiac and skeletal muscle cells. Front Biosci J Virtual Libr (2002) 7:d650–8. 10.2741/A801 11861217

[27] Zhao F Li P Chen SR Louis CF Fruen BR. Dantrolene inhibition of ryanodine receptor Ca^2+^ release channels. Molecular mechanism and isoform selectivity. J Biol Chem (2001) 276:13810–6. 10.1074/jbc.M006104200 11278295

[28] Meissner G Ryanodine receptor/Ca^2+^ release channels and their regulation by endogenous effectors. Annu Rev Physiol (1994) 56:485–508. 10.1146/annurev.ph.56.030194.002413 7516645

[29] Wieckowski MR Giorgi C Lebiedzinska M Duszynski J Pinton P. Isolation of mitochondria-associated membranes and mitochondria from animal tissues and cells. Nat Protoc (2009) 4:1582–90. 10.1038/nprot.2009.151 19816421

[30] Raturi A Simmen T. Where the endoplasmic reticulum and the mitochondrion tie the knot: the mitochondria-associated membrane (MAM). Biochim Biophys Acta (2013) 1833:213–24. 10.1016/j.bbamcr.2012.04.013 22575682

[31] Giorgi C Missiroli S Patergnani S Duszynski J Wieckowski MR Pinton P. Mitochondria-associated membranes: composition, molecular mechanisms, and physiopathological implications. Antioxid Redox Signal (2015) 22:995–1019. 10.1089/ars.2014.6223 25557408

[32] Szabadkai G Bianchi K Várnai P De Stefani D Wieckowski MR Cavagna D Chaperone-mediated coupling of endoplasmic reticulum and mitochondrial Ca^2+^ channels. J Cell Biol (2006) 175:901–11. 10.1083/jcb.200608073 17178908 PMC2064700

[33] Marchi S Patergnani S Pinton P. The endoplasmic reticulum-mitochondria connection: one touch, multiple functions. Biochim Biophys Acta (2014) 1837:461–9. 10.1016/j.bbabio.2013.10.015 24211533

[34] Foskett JK Philipson B The mitochondrial Ca^2+^ uniporter complex. J Mol Cell Cardiol (2015) 78:3–8. 10.1016/j.yjmcc.2014.11.015 25463276 PMC4307384

[35] Mallilankaraman K Doonan P Cárdenas C Chandramoorthy HC Müller M Miller R MICU1 is an essential gatekeeper for MCU-mediated mitochondrial Ca^2+^ uptake that regulates cell survival. Cell (2012) 151:630–44. 10.1016/j.cell.2012.10.011 23101630 PMC3486697

[36] Mallilankaraman K Cárdenas C Doonan PJ Chandramoorthy HC Irrinki KM Golenár T MCUR1 is an essential component of mitochondrial Ca^2+^ uptake that regulates cellular metabolism. Nat Cell Biol (2012) 14:1336–43. 10.1038/ncb2622 23178883 PMC3511605

[37] Raffaello A De Stefani D Sabbadin D Teardo E Merli G Picard A The mitochondrial calcium uniporter is a multimer that can include a dominant-negative pore-forming subunit. EMBO J (2013) 32:2362–76. 10.1038/emboj.2013.157 23900286 PMC3771344

[38] Hoffman NE Chandramoorthy HC Shanmughapriya S Zhang XQ Vallem S Doonan PJ SLC25A23 augments mitochondrial Ca^2+^ uptake, interacts with MCU, and induces oxidative stress-mediated cell death. Mol Biol Cell (2014) 25:936–47. 10.1091/mbc.E13-08-0502 24430870 PMC3952861

[39] Patron M Checchetto V Raffaello A Teardo E Vecellio Reane D Mantoan M MICU1 and MICU2 finely tune the mitochondrial Ca^2+^ uniporter by exerting opposite effects on MCU activity. Mol Cell (2014) 53:726–37. 10.1016/j.molcel.2014.01.013 24560927 PMC3988891

[40] Doonan PJ Chandramoorthy HC Hoffman NE Zhang X Cárdenas C Shanmughapriya S LETM1-dependent mitochondrial Ca^2+^ flux modulates cellular bioenergetics and proliferation. FASEB J (2014) 28:4936–49. 10.1096/fj.14-256453 25077561 PMC4200331

[41] Vais H Tanis JE Müller M Payne R Mallilankaraman K Foskett JK MCUR1, CCDC90A, is a regulator of the mitochondrial calcium uniporter. Cell Metab (2015) 22:533–5. 10.1016/j.cmet.2015.09.015 26445506 PMC5384258

[42] Vais H Mallilankaraman K Mak D-OD Hoff H Payne R Tanis JE EMRE is a matrix Ca^2+^ sensor that governs gatekeeping of the mitochondrial Ca^2+^ uniporter. Cell Rep (2016) 14:403–10. 10.1016/j.celrep.2015.12.054 26774479 PMC4731249

[43] Antony AN Paillard M Moffat C Juskeviciute E Correnti J Bolon B MICU1 regulation of mitochondrial Ca^2+^ uptake dictates survival and tissue regeneration. Nat Commun (2016) 7:10955 10.1038/ncomms10955 26956930 PMC4786880

[44] Tomar D Dong Z Shanmughapriya S Koch DA Thomas T Hoffman NE MCUR1 is a scaffold factor for the MCU complex function and promotes mitochondrial bioenergetics. Cell Rep (2016) 15:1673–85. 10.1016/j.celrep.2016.04.050 27184846 PMC4880542

[45] Rizzuto R Brini M Murgia M Pozzan T. Microdomains with high Ca^2+^ close to IP_3_-sensitive channels that are sensed by neighboring mitochondria. Science (1993) 262:744–7. 10.1126/science.8235595 8235595

[46] Csordás G Thomas AP Hajnóczky G. Quasi-synaptic calcium signal transmission between endoplasmic reticulum and mitochondria. EMBO J (1999) 18:96–108. 10.1093/emboj/18.1.96 9878054 PMC1171106

[47] Csordás G Várnai P Golenár T Roy S Purkins G Schneider TG Imaging interorganelle contacts and local calcium dynamics at the ER-mitochondrial interface. Mol Cell (2010) 39:121–32. 10.1016/j.molcel.2010.06.029 20603080 PMC3178184

[48] Booth DM Enyedi B Geiszt M Várnai P Hajnóczky G. Redox nanodomains are induced by and control calcium signaling at the ER-mitochondrial interface. Mol Cell (2016) 63:240–8. 10.1016/j.molcel.2016.05.040 27397688 PMC4998968

[49] Missiaen L De Smedt H Droogmans G Casteels R. Ca^2+^ release induced by inositol 1,4,5-trisphosphate is a steady-state phenomenon controlled by luminal Ca^2+^ in permeabilized cells. Nature (1992) 357:599–602. 10.1038/357599a0 1608471

[50] Pinton P Ferrari D Rapizzi E Di Virgilio F Pozzan T Rizzuto R The Ca^2+^ concentration of the endoplasmic reticulum is a key determinant of ceramide-induced apoptosis: significance for the molecular mechanism of Bcl-2 action. EMBO J (2001) 20:2690–701. 10.1093/emboj/20.11.2690 11387204 PMC125256

[51] Scorrano L Oakes SA Opferman JT Cheng EH Sorcinelli MD Pozzan T BAX and BAK regulation of endoplasmic reticulum Ca^2+^: a control point for apoptosis. Science (2003) 300:135–9. 10.1126/science.1081208 12624178

[52] Oakes SA Scorrano L Opferman JT Bassik MC Nishino M Pozzan T Proapoptotic BAX and BAK regulate the type 1 inositol trisphosphate receptor and calcium leak from the endoplasmic reticulum. Proc Natl Acad Sci U S A (2005) 102:105–10. 10.1073/pnas.0408352102 15613488 PMC544078

[53] Pinton P Ferrari D Magalhães P Schulze-Osthoff K Di Virgilio F Pozzan T Reduced loading of intracellular Ca^2+^ stores and downregulation of capacitative Ca^2+^ influx in Bcl-2-overexpressing cells. J Cell Biol (2000) 148:857–62. 10.1083/jcb.148.5.857 10704437 PMC2174537

[54] Bittremieux M Parys JB Pinton P Bultynck G ER functions of oncogenes and tumor suppressors: modulators of intracellular Ca^2+^ signaling. Biochim Biophys Acta (2016) 1863:1364–78. 10.1016/j.bbamcr.2016.01.002 26772784

[55] Bultynck G Kiviluoto S Henke N Ivanova H Schneider L Rybalchenko V The C terminus of Bax inhibitor-1 forms a Ca^2+^-permeable channel pore. J Biol Chem (2012) 287:2544–57. 10.1074/jbc.M111.275354 22128171 PMC3268414

[56] Kiviluoto S Schneider L Luyten T Vervliet T Missiaen L De Smedt H Bax inhibitor-1 is a novel IP_3_ receptor-interacting and -sensitizing protein. Cell Death Dis (2012) 3:e367 10.1038/cddis.2012.103 22875004 PMC3434651

[57] Høyer-Hansen M Bastholm L Szyniarowski P Campanella M Szabadkai G Farkas T Control of macroautophagy by calcium, calmodulin-dependent kinase kinase-beta, and Bcl-2. Mol Cell (2007) 25:193–205. 10.1016/j.molcel.2006.12.009 17244528

[58] Balaban RS The role of Ca^2+^ signaling in the coordination of mitochondrial ATP production with cardiac work. Biochim Biophys Acta (2009) 1787:1334–41. 10.1016/j.bbabio.2009.05.011 19481532 PMC3177847

[59] Territo PR Mootha VK French SA Balaban RS Ca^2+^ activation of heart mitochondrial oxidative phosphorylation: role of the F(0)/F(1)-ATPase. Am J Physiol Cell Physiol (2000) 278:C423–35. 10666039 10.1152/ajpcell.2000.278.2.C423

[60] Alers S Löffler AS Wesselborg S Stork B. Role of AMPK-mTOR-Ulk1/2 in the regulation of autophagy: cross talk, shortcuts, and feedbacks. Mol Cell Biol (2012) 32:2–11. 10.1128/MCB.06159-11 22025673 PMC3255710

[61] Kim J Kundu M Viollet B Guan K-L. AMPK and mTOR regulate autophagy through direct phosphorylation of Ulk1. Nat Cell Biol (2011) 13:132–41. 10.1038/ncb2152 21258367 PMC3987946

[62] Cárdenas C Foskett JK Mitochondrial Ca^2+^ signals in autophagy. Cell Calcium (2012) 52:44–51. 10.1016/j.ceca.2012.03.001 22459281 PMC3389293

[63] Decuypere J-P Bultynck G Parys JB A dual role for Ca^2+^ in autophagy regulation. Cell Calcium (2011) 50:242–50. 10.1016/j.ceca.2011.04.001 21571367

[64] Williams A Hayashi T Wolozny D Yin B Su T-C Betenbaugh MJ The non-apoptotic action of Bcl-xL: regulating Ca^2+^ signaling and bioenergetics at the ER-mitochondrion interface. J Bioenerg Biomembr (2016) 48:211–25. 10.1007/s10863-016-9664-x 27155879 PMC6737942

[65] White C Li C Yang J Petrenko NB Madesh M Thompson CB The endoplasmic reticulum gateway to apoptosis by Bcl-XL modulation of the InsP_3_R. Nat Cell Biol (2005) 7:1021–8. 10.1038/ncb1302 16179951 PMC2893337

[66] Sano R Hou Y-CC Hedvat M Correa RG Shu C-W Krajewska M Endoplasmic reticulum protein BI-1 regulates Ca^2+^-mediated bioenergetics to promote autophagy. Genes Dev (2012) 26:1041–54. 10.1101/gad.184325.111 22588718 PMC3360560

[67] Cárdenas C Müller M McNeal A Lovy A Jaňa F Bustos G Selective vulnerability of cancer cells by inhibition of Ca^2+^ transfer from endoplasmic reticulum to mitochondria. Cell Rep (2016) 14:2313–24. 10.1016/j.celrep.2016.02.030 26947070 PMC4794382

[68] Singh A Chagtoo M Tiwari S George N Chakravarti B Khan S Inhibition of inositol 1, 4, 5-trisphosphate receptor induce breast cancer cell death through deregulated autophagy and cellular bioenergetics. J Cell Biochem (2017). 10.1002/jcb.25891 28106298

[69] Wiel C Lallet-Daher H Gitenay D Gras B Le Calvé B Augert A Endoplasmic reticulum calcium release through ITPR2 channels leads to mitochondrial calcium accumulation and senescence. Nat Commun (2014) 5:3792. 10.1038/ncomms4792 24797322

[70] Li C Wang X Vais H Thompson CB Foskett JK White C Apoptosis regulation by Bcl-x(L) modulation of mammalian inositol 1,4,5-trisphosphate receptor channel isoform gating. Proc Natl Acad Sci U S A (2007) 104:12565–70. 10.1073/pnas.0702489104 17636122 PMC1941509

[71] Missiroli S Bonora M Patergnani S Poletti F Perrone M Gafà R PML at mitochondria-associated membranes is critical for the repression of autophagy and cancer development. Cell Rep (2016) 16:2415–27. 10.1016/j.celrep.2016.07.082 27545895 PMC5011426

[72] Giorgi C Ito K Lin H-K Santangelo C Wieckowski MR Lebiedzinska M PML regulates apoptosis at endoplasmic reticulum by modulating calcium release. Science (2010) 330:1247–51. 10.1126/science.1189157 21030605 PMC3017677

[73] Rong Y-P Aromolaran AS Bultynck G Zhong F Li X McColl K Targeting Bcl-2-IP_3_ receptor interaction to reverse Bcl-2’s inhibition of apoptotic calcium signals. Mol Cell (2008) 31:255–65. 10.1016/j.molcel.2008.06.014 18657507 PMC3660092

[74] Rong Y-P Bultynck G Aromolaran AS Zhong F Parys JB De Smedt H The BH4 domain of Bcl-2 inhibits ER calcium release and apoptosis by binding the regulatory and coupling domain of the IP_3_ receptor. Proc Natl Acad Sci U S A (2009) 106:14397–402. 10.1073/pnas.0907555106 19706527 PMC2728114

[75] Raturi A Gutiérrez T Ortiz-Sandoval C Ruangkittisakul A Herrera-Cruz MS Rockley JP TMX1 determines cancer cell metabolism as a thiol-based modulator of ER-mitochondria Ca^2+^ flux. J Cell Biol (2016) 214:433–44. 10.1083/jcb.201512077 27502484 PMC4987292

[76] Krols M Bultynck G Janssens S. ER-mitochondria contact sites: a new regulator of cellular calcium flux comes into play. J Cell Biol (2016) 214:367–70. 10.1083/jcb.201607124 27528654 PMC4987300

[77] Giorgi C Bonora M Sorrentino G Missiroli S Poletti F Suski JM p53 at the endoplasmic reticulum regulates apoptosis in a Ca^2+^-dependent manner. Proc Natl Acad Sci U S A (2015) 112:1779–84. 10.1073/pnas.1410723112 25624484 PMC4330769

[78] Giorgi C Bonora M Missiroli S Poletti F Ramirez FG Morciano G Intravital imaging reveals p53-dependent cancer cell death induced by phototherapy via calcium signaling. Oncotarget (2015) 6:1435–45. 10.18632/oncotarget.2935 25544762 PMC4359305

[79] Madreiter-Sokolowski CT Gottschalk B Parichatikanond W Eroglu E Klec C Waldeck-Weiermair M Resveratrol specifically kills cancer cells by a devastating increase in the Ca^2+^ coupling between the greatly tethered endoplasmic reticulum and mitochondria. Cell Physiol Biochem (2016) 39:1404–20. 10.1159/000447844 27606689 PMC5382978

[80] Huang H Shah K Bradbury NA Li C White C. Mcl-1 promotes lung cancer cell migration by directly interacting with VDAC to increase mitochondrial Ca^2+^ uptake and reactive oxygen species generation. Cell Death Dis (2014) 5:e1482. 10.1038/cddis.2014.419 25341036 PMC4237246

[81] Tosatto A Sommaggio R Kummerow C Bentham RB Blacker TS Berecz T The mitochondrial calcium uniporter regulates breast cancer progression via HIF-1α. EMBO Mol Med (2016) 8:569–85. 10.15252/emmm.201606255 27138568 PMC4864890

[82] Doghman-Bouguerra M Granatiero V Sbiera S Sbiera I Lacas-Gervais S Brau F FATE1 antagonizes calcium- and drug-induced apoptosis by uncoupling ER and mitochondria. EMBO Rep (2016) 17:1264–80. 10.15252/embr.201541504 27402544 PMC5007562

[83] Naon D Scorrano L. At the right distance: ER-mitochondria juxtaposition in cell life and death. Biochim Biophys Acta (2014) 1843:2184–94. 10.1016/j.bbamcr.2014.05.011 24875902

[84] Giorgi C Baldassari F Bononi A Bonora M De Marchi E Marchi S Mitochondrial Ca^2+^ and apoptosis. Cell Calcium (2012) 52:36–43. 10.1016/j.ceca.2012.02.008 22480931 PMC3396846

[85] Zhivotovsky B Orrenius S. Calcium and cell death mechanisms: a perspective from the cell death community. Cell Calcium (2011) 50:211–21. 10.1016/j.ceca.2011.03.003 21459443

[86] De Stefani D Bononi A Romagnoli A Messina A De Pinto V Pinton P VDAC1 selectively transfers apoptotic Ca^2+^ signals to mitochondria. Cell Death Differ (2012) 19:267–73. 10.1038/cdd.2011.92 21720385 PMC3263501

[87] Zheng Y Shen X. H_2_O_2_ directly activates inositol 1,4,5-trisphosphate receptors in endothelial cells. Redox Rep (2005) 10:29–36. 10.1179/135100005X21660 15829109

[88] Marchi S Rimessi A Giorgi C Baldini C Ferroni L Rizzuto R Akt kinase reducing endoplasmic reticulum Ca^2+^ release protects cells from Ca^2+^-dependent apoptotic stimuli. Biochem Biophys Res Commun (2008) 375:501–5. 10.1016/j.bbrc.2008.07.153 18723000 PMC2576286

[89] Szado T Vanderheyden V Parys JB De Smedt H Rietdorf K Kotelevets L Phosphorylation of inositol 1,4,5-trisphosphate receptors by protein kinase B/Akt inhibits Ca^2+^ release and apoptosis. Proc Natl Acad Sci U S A (2008) 105:2427–32. 10.1073/pnas.0711324105 18250332 PMC2268153

[90] Rimessi A Marchi S Fotino C Romagnoli A Huebner K Croce CM Intramitochondrial calcium regulation by the FHIT gene product sensitizes to apoptosis. Proc Natl Acad Sci U S A (2009) 106:12753–8. 10.1073/pnas.0906484106 19622739 PMC2722368

[91] Pinton P Giorgi C Siviero R Zecchini E Rizzuto R. Calcium and apoptosis: ER-mitochondria Ca^2+^ transfer in the control of apoptosis. Oncogene (2008) 27:6407–18. 10.1038/onc.2008.308 18955969 PMC2844952

[92] Bonora M Giorgi C Pinton P. Novel frontiers in calcium signaling: a possible target for chemotherapy. Pharmacol Res (2015) 99:82–5. 10.1016/j.phrs.2015.05.008 26028552

[93] Florea A-M Varghese E McCallum JE Mahgoub S Helmy I Varghese S Calcium-regulatory proteins as modulators of chemotherapy in human neuroblastoma. Oncotarget (2017) 8:22876–93. 10.18632/oncotarget.15283 28206967 PMC5410270

[94] Hwang M-S Schwall CT Pazarentzos E Datler C Alder NN Grimm S Mitochondrial Ca^2+^ influx targets cardiolipin to disintegrate respiratory chain complex II for cell death induction. Cell Death Differ (2014) 21:1733–45. 10.1038/cdd.2014.84 24948011 PMC4211371

[95] Galluzzi L. Novel insights into PML-dependent oncosuppression. Trends Cell Biol (2016) 26:889–90. 10.1016/j.tcb.2016.09.001 27663133

[96] Kasahara A Scorrano L. Mitochondria: from cell death executioners to regulators of cell differentiation. Trends Cell Biol (2014) 24:761–70. 10.1016/j.tcb.2014.08.005 25189346

[97] Cogliati S Enriquez JA Scorrano L. Mitochondrial cristae: where beauty meets functionality. Trends Biochem Sci (2016) 41:261–73. 10.1016/j.tibs.2016.01.001 26857402

[98] Cereghetti GM Stangherlin A Martins de Brito O Chang CR Blackstone C Bernardi P Dephosphorylation by calcineurin regulates translocation of Drp1 to mitochondria. Proc Natl Acad Sci U S A (2008) 105:15803–8. 10.1073/pnas.0808249105 18838687 PMC2572940

[99] Morciano G Giorgi C Balestra D Marchi S Perrone D Pinotti M Mcl-1 involvement in mitochondrial dynamics is associated with apoptotic cell death. Mol Biol Cell (2016) 27:20–34. 10.1091/mbc.E15-01-0028 26538029 PMC4694758

[100] Morciano G Pedriali G Sbano L Iannitti T Giorgi C Pinton P. Intersection of mitochondrial fission and fusion machinery with apoptotic pathways: role of Mcl-1. Biol Cell (2016) 108:279–93. 10.1111/boc.201600019 27234233

[101] Zorov DB Juhaszova M Sollott SJ. Mitochondrial ROS-induced ROS release: an update and review. Biochim Biophys Acta (2006) 1757:509–17. 10.1016/j.bbabio.2006.04.029 16829228

[102] Guo JY Teng X Laddha SV Ma S Van Nostrand SC Yang Y Autophagy provides metabolic substrates to maintain energy charge and nucleotide pools in Ras-driven lung cancer cells. Genes Dev (2016) 30:1704–17. 10.1101/gad.283416.116 27516533 PMC5002976

[103] Lashinger LM O’Flanagan CH Dunlap SM Rasmussen AJ Sweeney S Guo JY Starving cancer from the outside and inside: separate and combined effects of calorie restriction and autophagy inhibition on Ras-driven tumors. Cancer Metab (2016) 4:18. 10.1186/s40170-016-0158-4 27651895 PMC5025535

[104] White E Mehnert JM Chan CS Autophagy, metabolism, and cancer. Clin Cancer Res (2015) 21:5037–46. 10.1158/1078-0432.CCR-15-0490 26567363 PMC4646728

[105] Bultynck G. Onco-IP_3_Rs feed cancerous cravings for mitochondrial Ca^2+^. Trends Biochem Sci (2016) 41:390–3. 10.1016/j.tibs.2016.03.006 27068804

[106] Mitra K Wunder C Roysam B Lin G Lippincott-Schwartz J. A hyperfused mitochondrial state achieved at G1-S regulates cyclin E buildup and entry into S phase. Proc Natl Acad Sci U S A (2009) 106:11960–5. 10.1073/pnas.0904875106 19617534 PMC2710990

[107] Hochegger H Takeda S Hunt T. Cyclin-dependent kinases and cell-cycle transitions: does one fit all? Nat Rev Mol Cell Biol (2008) 9:910–6. 10.1038/nrm2510 18813291

[108] Finkel T Hwang PM The Krebs cycle meets the cell cycle: mitochondria and the G1-S transition. Proc Natl Acad Sci U S A (2009) 106:11825–6. 10.1073/pnas.0906430106 19617546 PMC2715508

[109] Chang GG Huang TM Wang JK Lee HJ Chou WY Meng CL. Kinetic mechanism of the cytosolic malic enzyme from human breast cancer cell line. Arch Biochem Biophys (1992) 296:468–73. 10.1016/0003-9861(92)90599-R 1632639

[110] Roderick HL Cook SJ. Ca^2+^ signalling checkpoints in cancer: remodelling Ca^2+^ for cancer cell proliferation and survival. Nat Rev Cancer (2008) 8:361–75. 10.1038/nrc2374 18432251

[111] Bernard D Wiel C Transport and senescence. Oncoscience (2015) 2:741–2. 10.18632/oncoscience.191 26501075 PMC4606003

[112] Ziegler DV Wiley CD Velarde MC. Mitochondrial effectors of cellular senescence: beyond the free radical theory of aging. Aging Cell (2015) 14:1–7. 10.1111/acel.12287 25399755 PMC4310776

[113] Bittremieux M Bultynck G p53 and Ca^2+^ signaling from the endoplasmic reticulum: partners in anti-cancer therapies. Oncoscience (2015) 2:233–8. 10.18632/oncoscience.139 25897426 PMC4394128

[114] van Delft MF Wei AH Mason KD Vandenberg CJ Chen L Czabotar PE The BH3 mimetic ABT-737 targets selective Bcl-2 proteins and efficiently induces apoptosis via Bak/Bax if Mcl-1 is neutralized. Cancer Cell (2006) 10:389–99. 10.1016/j.ccr.2006.08.027 17097561 PMC2953559

[115] Oltersdorf T Elmore SW Shoemaker AR Armstrong RC Augeri DJ Belli BA An inhibitor of Bcl-2 family proteins induces regression of solid tumours. Nature (2005) 435:677–81. 10.1038/nature03579 15902208

[116] Xie Q Su J Jiao B Shen L Ma L Qu X ABT737 reverses cisplatin resistance by regulating ER-mitochondria Ca^2+^ signal transduction in human ovarian cancer cells. Int J Oncol (2016) 49:2507–19. 10.3892/ijo.2016.3733 27748803

[117] Dadi PK Ahmad M Ahmad Z. Inhibition of ATPase activity of *Escherichia coli* ATP synthase by polyphenols. Int J Biol Macromol (2009) 45:72–9. 10.1016/j.ijbiomac.2009.04.004 19375450

[118] Luyten T Welkenhuyzen K Roest G Kania E Wang L Bittremieux M Resveratrol-induced autophagy is dependent on IP_3_Rs and on cytosolic Ca^2+^. Biochim Biophys Acta (2017). 10.1016/j.bbamcr.2017.02.013 28254579

